# Older Adults’ Gender, Age and Physical Activity Effects on Anxiety, Optimism, Resilience and Engagement

**DOI:** 10.3390/ijerph17207561

**Published:** 2020-10-17

**Authors:** Alfonso Martínez-Moreno, Ricardo José Ibáñez-Pérez, Francisco Cavas-García, Francisco Cano-Noguera

**Affiliations:** INGESPORT Research Group, Department of Physical Activity and Sports, University of Murcia, 30720 Santiago de la Ribera-San Javier, Spain; ricardojose.ibanez@um.es (R.J.I.-P.); francisco.cavas@um.es (F.C.-G.); francisco.cano@um.es (F.C.-N.)

**Keywords:** health, physical activity, successful aging

## Abstract

The purpose of the study was to understand the effects of gender and age on anxiety, optimism, resilience and engagement in a group of older adults. An observational, quantitative, descriptive and transversal design was used with non-probabilistic sampling. Descriptive statistical analyses, reliability tests (Cronbach’s alpha) and linear correlation tests (Pearson’s) were performed, and the development of multivariate linear regression models was conducted. Female participants in the sample had higher levels in anxiety and pessimism, while male participants scored higher in optimism, engagement and resilience. Participants who practiced physical activity (PA) had better scores in optimism, engagement and resilience. The sample comprised 55.1% men and 44.9% women, between the ages of 51 and 93, with an average of 68.1 years, all participants completed the questionnaire Anxiety Scale-2 (SAS-2) the Revised Life Orientation Test (LOT-R) the short version of the Utrecht Work Engagement Scale questionnaire (UWES-9) the short version of the CD-RISC. As for marital status, there were significant differences between single participants and romantic partner. Singles participants showed higher levels of anxiety than their married counterparts, while those in a relationship scored higher in optimism, engagement and resilience. The model was statistically significant *F* (9;352) = 14.6; *p* < 0.001, explaining 27% of the variance in optimism. The data indicated that PA practice and living with a partner in an inland area is associated with less anxiety, which may have implications for programs and activities designed for older adults.

## 1. Introduction

Spain’s population is continuing to age, with 9,057,193 older people (more than 65 years old) living in Spain and a life expectancy of 83.2 years as of 1 January 2019, according to the National Institute of Statistics. The region of Murcia is one of the least-aged autonomous communities in Spain, with only 16% of the population being. In relation to the high life expectancy of people aged 65 and over, women generally live longer than men, 23.5 out of 19.5 years [1]. Older adults (OAs) are recognized as a risk group, due to the psychological, emotional and psychosocial changes that occur in the later stage of life [2]. Rowe and Kahn [3] defined successful aging as the interrelationship of: (i) low probability of disease and disease-related disabilities, (ii) high cognitive and physical functioning and (iii) commitment to life. The OAs focus their concerns on health and family [4], although health problems and physical limitations can encourage participation in active leisure activities among adults, over 65 years old [5]. The most common mental disorders found in OAs are anxiety, affective and substance use disorders [6].

It is estimated that between 1.2 and 17.2% of the elderly population suffers from an anxiety disorder [7]. Anxiety is a transitory emotional state, fostered by feelings of subjective tension and apprehension, implemented in basic psychological processes such as attention, perception, memory, emotion and thought [8]. It has been considered in all previous research as something negative [9]. It often grows or is accentuated with age, or as a result of the organic modifications that occur during aging, without becoming pathological [10]. In OAs, anxiety can have negative consequences for physical and emotional health, but this age group rarely seeks help for such symptoms and professionals often pay little attention to them [11,12].

Particularly in the last decade, positive psychology has led research to focus on strengths and optimal functioning rather than weaknesses and dysfunction. [13,14]. Positive psychology has been defined as the “scientific study of optimal human functioning” [15]. Within this context, variables have been studied that are related to people’s well-being, particularly optimism [16]. According to positive psychology, intelligent optimism is the ability to face adversities as challenges rather than threats, turning crises into opportunities [17] and further enhancing people’s abilities, rather than restructuring the capabilities which one lacks [18]. Optimism thus protects a person against adversities [17,19].

Resilience is another effective skill, as it facilitates the way to overcome and cope with the negative effects of risk exposure, stressful experiences and negative dynamics associated with risk [20,21,22]. A person’s assessment of a situation that he or she considers stressful or threatening depends on the person’s personal ability to deal with such situations effectively [23,24]. Resilient people face adversity, becoming reinforced by such situations [25]. In old age, resilience is systematically associated with perceived optimal functioning in some areas such as: enjoying good health, mobility, physical and cognitive functioning, as well as maintaining social networks and support [26].

Engagement is a motivational construct, including components of activation, energy, effort and persistence [27]. It is fundamental to promoting both good health and well-being [28]. Engagement is relevant because it gives characteristics and positive aspects, as well as providing optimism and self-esteem in the individual [29]. It also enables the predisposition of a good mental state, with higher levels of energy and physical and mental persistence [30]. A higher level of engagement tends to improve the quality of life [31]. There are several indicators that positively relate engagement with health, related to low level of stress [32].

Physical activity seems to be associated with a better ability to cope with stress [33], as well as a decrease in burnout symptoms [34], reduction in anxiety symptoms [35] and even absence of anxiety [36]. Also, PA is a guarantee of improvement of functional health and quality of life in older adults [37], its practice is closely related to health and quality of life benefits [38].

Individually, anxiety [10], optimism [17,18,19], resilience [25] and engagement [28] have been studied in this age group. However, there is no study, known to the authors, that analyzes these variables at the same time. The reason for analyzing the above variables together is to be able to know how they interact with each other in a particular population group. It is known that optimism acts as a mediator of anxiety [39,40], that resilience precedes and contributes directly and negatively to anxiety [41,42,43] and that resilience is also a modulator in stress processes in general [44]. Resilience is related to optimism and engagement [45]. This leads us to consider the present study, to find out how these interrelations behave in this age group. Previous studies have analyzed anxiety, optimism, resilience and engagement individually, but none have analyzed the variables together in OAs. The main objective of the present research is thus the influences of anxiety, optimism, resilience and engagement in older adults, overall and when grouped by gender or age. The specific objectives of this study were:

(i) To determine whether there are differences between OAs who practice physical activity (PA) and those who do not;
(ii) To observe any differences between participants who have a romantic partner and those who are separated, single or widowed;
(iii) To check whether being unemployed and/or retired has an influence on PA;
(iv) To determine whether age and gender influence anxiety, optimism, resilience and engagement.

Older adults, like any other age group, require specific studies that analyze their peculiarities and provide solutions.

Thus, we propose the following hypotheses: (h1) older people are more anxious, (h2) women reach higher values in anxiety than men, (h3) physical activity dampens anxiety, (h4) living with a partner mitigates anxiety, (h5) women are more resilient than men, (h6) optimism is positively related to resilience.

## 2. Materials and Methods

Next, we will develop the materials and methods used in the study; for a better understanding, we have included different sections.

### 2.1. Design and Participants

For the purposes of this research, a quantitative, descriptive and transversal study design was used with non-probabilistic sampling [46]; it was conducted in the places where the managers and users agreed to participate in the study. The target population of the study was 233,300 subjects (data as of 31 December 2019, according to the National Institute of Statistics). A total of 395 surveys were collected, of which 14 (3.5%) were eliminated after applying criteria to minimize bias of minimum effort and consent, resulting in a total of 381 valid surveys. Considering these data, a sampling error of 5.02% at a 95% confidence level was determined. Participants were between the ages of 51 and 93 with an average age of 68.1 years (SD = 8.6). According to the WHO, older adults are considered to be from the age of 65, but people from the age of 51 have been included in this study, as they were pre-retired.

### 2.2. Procedure

Participants were approached directly on the street at the entrances and exits of the centers, as well as through the managers and directors of the different institutions (i.e., elderly centers, social centers, sports centers). They were informed of the objectives of the study and asked for their cooperation. Once consent was obtained, either by the center or individually, the objectives of the research were explained to them, and they were assured of the anonymity of their data and the voluntary nature of their participation. Those who agreed to participate filled out the informed consent form and the questionnaire. Those OAs who voluntarily agreed to participate in the study and were autonomous in their daily lives—dressing, grooming, moving around town—were included. Those who could not perform any of the above functions were excluded. A member of the research group was present at all times, applying the protocol established for the investigation and resolving any issues that arose. Each participant was given 15 to 20 min to complete the questionnaire. The study was carried out under the guidelines of the Helsinki Declaration and the design did not contain any ethical aspects requiring prior authorization from the Bioethics Committee of the University of Murcia, Spain.

### 2.3. Instrument

To observe the anxiety, the Sport Anxiety Scale-2 [47], adapted into Spanish by Ramis et al. [48], was used. The questionnaire contained 15 items divided into three subscales in order to detect individual differences: Somatic Anxiety (A-S), Concern (A-P) and Deconcentration (A-D). An overall anxiety (A-T) score was obtained from the summation of all of the items. Each response was given on a four-point Likert scale, ranging from 1 (nothing) to 4 (a lot), (i.e., “I feel my muscles tense because I am nervous”). Total scores per subscale were obtained from the sum of the scores of the items and could range from 5 to 20, where a low score would indicate a low probability of anxiety and a high score would indicate a tendency to present anxiety in a competition situation. The total score, based on the sum of all of the items, had a potential range between 15 and 60. In the sample of this study, the questionnaire showed good internal consistency as a whole (⍺ = 0.88), as well as for the A-S (⍺ = 0.80), A-P (⍺ = 0.83) and A-D (⍺ = 0.81) dimensions.

The Revised Life Orientation Test (LOT-R) by Scheier [49], adapted into Spanish by Otero et al. [50], was used to measure the level of dispositional optimism or a widespread predisposition towards expectations of positive results. It is the most widely used instrument to measure this construct [51,52]. It is comprised of 10 items in which subjects indicated their degree of agreement or disagreement with statements such as “I am always optimistic about the future” on a five-point Likert scale, where 0 meant “strongly disagree” and 4 meant “strongly agree”. Four of the items were control or filler items, designed to make the contents of the instrument less obvious [53], and of the remaining items, three were positive (optimistic) and three were negative (pessimistic). Regarding the correction and interpretation of the test, two options appear, one by Ferrando et al. [51], where the measurement of (optimism and pessimism) is made separately—the option chosen for this investigation—and, on the other hand, the measurement of total optimism by reversing the items written in the negative direction. The different factorial works supported obtaining the two factors (optimism and pessimism) [54,55]. In the analyzed sample, the reliability of the total LOT-R scale was ⍺ = 0.82. LOT-O, which measured optimism (⍺ = 0.80), and LOT-P, which measured pessimism (⍺ = 0.83), were also fairly reliable.

For the assessment of engagement, the short version of the Utrecht Work Engagement Scale questionnaire (UWES-9) by Schaufeli and Bakker [56] was applied, consisting of nine items and three dimensions or factors: vigor, dedication and absorption. The score for each item ranged from 0 (never) to 6 (always) (i.e., “When I wake up in the morning, I feel like going to the activities”). The total engagement value was obtained by calculating the sum of the scores of all of the items, divided by the number of dimensions. The average scores of the three dimensions of UWES-9 were obtained by adding the scores of each subscale and dividing its result by the number of items in the respective subscale. Therefore, the UWES-9 could yield three partial scores, corresponding to each subscale, and a total score within the range of 0 and 6 points. In the sample analyzed, the questionnaire presented good internal consistency for the whole questionnaire (UWES-T), ⍺ = 0.81 and for both vigor (UWES-V), ⍺ = 0.80, and for absorption (UWES-A), ⍺ = 0.80. Only the dimension of dedication (UWES-D) showed acceptable reliability ⍺ = 0.79).

Resilience was evaluated using the short version of the CD-RISC questionnaire, translated into Spanish by Notary-Pacheco [57], consisting of 10 items from the original Connor and Davidson scale [58]. Participants were asked to respond about the extent to which they agreed with each of the items (i.e., “I can keep the concentration under pressure”) on a five-point Likert scale ranging from 0 (“totally in disagreement”) to 4 (“totally agreed”). In the sample analyzed, the CD-RISC questionnaire had a reliability of ⍺ = 0.81, being quite reliable.

### 2.4. Statistical Analysis

For the descriptive statistical analysis of the sample, the number of cases presented in each category and the corresponding percentage for the qualitative variables were obtained. For the quantitative variables, the minimum, maximum, mean and standard deviation (SD) values were obtained. Cronbach’s alpha was calculated in order to check the reliability of the different scales in this particular sample, and correlations between variables were calculated using the Pearson linear correlation coefficient (r). For the quantitative variables, the Student *t*-test was performed for mean comparisons between two groups, and for comparisons of more than two groups, an analysis of variance (ANOVA) was performed. Assumptions of normality and uniformity of the variances required for mean comparisons were tested using the Kolmogorov–Smirnov test and the Levene test, respectively. Finally, a multivariate linear regression model was developed to determine the possible effects of the variables sex, age, place of residence (littoral or inland), the type of center (none, private or public) and the practice of PA, as well as the scales UWES, SAS-2 and resilience scales in LOT-R. The effect size was measured by partial eta2 (eta2) and Cohen’s D according to the statistical test performed, considering the ranges (0.2 small; 0.5 medium and 0.8 high). Statistical analyses were performed using the SPSS 25.0 program for Windows (IBM, New York, USA). Statistical significance was defined as *p* < 0.05.

## 3. Results

The sample consisted of 210 (55.1%) males and 171 (44.9%) females. Over two-thirds of participants lived with a partner (72.4%) and 105 (27.6%) lived alone. In terms of education, 34.4% of the sample had no level of education, 31.5% had a basic level of education, 18.9% had obtained a Bachelor’s degree and 15.2% had completed university studies. Over half of the participants (64.5%) are retired, 7.4% unemployed and the remaining 28.1%, was working. Two-thirds of participants did not attend any type of center (61.4%), while 8.7% attended a private center and the remaining 29.7% attended a public center. Of the total sample, 83.7% were not institutionalized (i.e., they lived independently) and 16.3% lived in an institution or residence for OAs. Two-thirds of the sample (69.3%) practiced PA and the remaining 30.7% did not. Nearly half of the participants (43.6%) practiced PA more than three days a week, 15.2% practiced three days a week, 34.1% practiced two days a week and 7.2% practiced only a single day every week.

The means, standard deviations, Cronbach’s alpha reliability indices and the correlations between the different scales used in the research have been presented in Table 1. The internal consistency rates were all greater than 0.80, indicating strong reliability except for in the UWES-D dimension, which showed acceptable reliability (⍺ = 0.79). The association between A-T and A-S, A-P and A-D was strong significant and positive, the association with LOT-P was weak. AT also correlated negatively and weakly with LOT-R, LOT-O, UWES-T, UWES-V, UWES-D, UWES-A and resilience. With regard to A-S, it was positively and statistically significantly associated with A-P and A-D, although only moderately, and with LOT-P, although the relationship was weak. Relationships with the other scales and dimensions were also present, but all negative and weak.

In terms of AP, it was positively and statistically significantly related to A-D and LOT-P, negatively related to LOT-R and LOT-O, and showing no significant relationships with the remaining scales and dimensions. All of the significant associations were low. There was a low positive and significant relationship between A-D and LOT-P, and there was a similar negative relationship between A-D and the remaining scales and variables. Regarding LOT-R, there were strong positive correlations with LOT-O and low correlations with UWES-T, UWES-V, UWES-D and UWES-A. LOT-R showed a moderate correlation with resilience and a strong negative correlation with LOT-P. With regard to LOT-O, LOT-R had a low positive and significant association with UWES-V and UWES-A, a moderate relationship with UWES-T, UWES-D and resilience, and a negative relationship with LOT-P. The data showed a low and negative association between LOT-P and UWES-T, UWES-V, UWES-D and resilience and no relationship with the remaining scales and dimensions. Regarding UWES-T, it had a very strong and positive significant relationship with UWES-V, UWES-D and UWES-A, and a moderate relationship with resilience. UWES-V was highly positively and significantly associated with UWES-D and UWES-A and moderately associated with resilience. Regarding UWES-D, there was a strong, positive and significant relationship with UWES-A and a moderate relationship with Resilience. There was also a moderate relationship between UWES-A and resilience.

The results of each scale and the results of the Student *t*-tests performed to identify statistically significant differences between males and females have been organized by gender and presented in the Table 2. Female participants had higher levels of A-T, A-S, A-P, A-D, as well as LOT-P than men. Males had higher scores in LOT-R, LOT-O, UWES-T, UWES-V, UWES-D, UWES-A, as well as resilience. There were statistically significant differences between males and females in A-T, A-S and A-D, but there were no differences between the remaining variables and dimensions.

Regarding age, Pearson’s correlation coefficient, Table 3, showed a statistically significant positive relationship between age and A-T, A-S, A-S and A-D, meaning that older participants had higher total anxiety, somatic anxiety and deconcentration. There was also a significant, but negative relationship between age and UWES-A, with a reduction in absorption as the number of years increased. The other scales and dimensions did not show any significant relationships with age.

When comparing the scales according to whether participants practiced PA or not, the results revealed that participants who practiced PA reached higher scores in AD, LOT-T, LOT-O, LOT-P, UWES-T, as well as in resilience. In contrast, those who did not practice PA scored higher in AT and AS. There were statistically significant differences in LOT-R (*p* = 0.048), LOT-O (*p* = 0.015), UWES-T (*p* < 0.001), UWES-V (*p* < 0.001), UWES-D (*p* < 0.001), UWES-A (*p* < 0.001) and resilience (*p* = 0.016).

In relation to the number of days of PA practice, Pearson’s correlation coefficient showed a statistically significant, weak negative relationship in terms of A-T (*r* = −0.289, *p* = 0.002), A-S (*r* = −0.342, *p* = 0.022), A-P (*r* = −0.256, *p* = 0.011), A-D (*r* = −0.397, *p* = 0.001) and LOT-P (*r* = −0.359, *p* ≤ 0.001), and a weak positive relationship with LOT-R (*r* = −0.135, *p* = 0.033). The other scales showed no significant relationships with the number of days of PA practice.

In terms of marital status, the data showed that single participants attained higher scores in A-T, A-S, A-P, A-D and LOT-P, while participants living with a partner attained higher scores in LOT-R, LOT-O, UWES-T, UWES-V, UWES-D and resilience. UWES-A had identical results in both groups. There were statistically significant differences between the two groups with regard to A-T (*p* = 0.001), A-S (*p* = 0.001), A-P (*p* = 0.023) and A-D (*p* ≤ 0.001). The other scales showed no significant differences between participants living with a partner and single participants.

When analyzing the sample under study, OAs living inland attained higher scores in A-T, A-S, A-P, LOT-R, LOT-O, UWES-T, UWES-V, UWES-D, UWES-A and resilience, while those living on the coast scored higher in A-D and LOT-P. There were significant differences in LOT-R in terms of A-S (*p* = 0.044) and UWES-V (*p* = 0.025) between those living inland and those living on the coast. The remaining scales did not show any significant relationships.

In relation to occupation (retired, unemployed, working) of the sample participants, the ANOVA showed statistically significant differences between groups on the AT scale. Participants who are working had significantly lower scores in A-T (*p* = 0.011) and A-D (*p* ≤ 0.001) than those who were standing or retired. The remaining scales showed no significant differences between occupation statuses.

The participants were grouped into <65 (39.9%), 65−75 (39.1%) and >75 (21%) years and compared in relation to the scales (see Table 4). It was noted that there were significant differences (*p* ≤ 0.001) between the three groups in terms of A-T and A-D. A-S was significant (*p* = 0.021) among the participants aged <65 and >75, while UWES-A showed significant differences (*p* = 0.048) among the groups aged <65 and 65–75 in relation to >75, presenting these higher values.

To determine the effect of the variables sex and age, place of residence, type of center, Table 5, PA practice and in each of the scales, A-T, LOT-R, UWES-T and resilience, a linear regression was performed. The results are shown in Table 4. The model was statistically significant *F* (9;352) = 14.6, *p* < 0.001, explaining 27% of the explanatory variance. In terms of the scales, A-T and resilience showed significant effects in such a way that higher A-T values were associated with lower LOT-R values, while higher values in resilience were associated with higher values in LOT-R. Age and sex had no effect on the other variables.

## 4. Discussion

After presenting the data, we will now answer each of the hypotheses put forward. In relation to (h1), older people are more anxious, it is confirmed, since the results of the study showed that, since the results of the SAS-2 scale are generally higher in OAs than in other age groups, coinciding with the results of González et al.’s study [59], this found that older people showed a higher cut-off point than other age groups on the SAS-2 scale. Participants show significant differences in relation to the three age groups (<65; 65–75 and >75 years) with respect to total anxiety and deconcentration anxiety, in the line of Vitores [60]. Similarly, Wisocki [61] concluded that, although older people had more problems (e.g., illnesses, functional limitations, cognitive deficits), they worried less than younger people.

As for (h2), women reach higher values of anxiety than men, it is ratified, coincided with the other studies that found females to have greater anxiety than men [62,63,64,65,66,67], which is worrying because studies, such as Granados-Ramos et al. [68], have shown that cases of severe depression were associated with anxiety.

With regard to (h3), physical activity reduces anxiety, the hypothesis is also demonstrated, since the participants who practiced PA generally demonstrated higher levels of resilience, coinciding with an essential finding in Cortés et al.’s study [69]: the beneficial effects of PA on resilience. These results reinforced the importance of non-pharmacological interventions and the effectiveness of other types of activities in improving psychological symptoms [70]. Cortes et al. [69] observed a high degree of resilience, both in males and females who were in a relationship, which was replicated in the findings from this study. The resilience levels of the OAs in this study did not vary depending on where they lived, which is consistent with Wells’ study [70], which investigated the levels of resilience in rural, suburban and urban areas.

In relation to (h4), living with a partner mitigates anxiety, it is corroborated in that single participants had higher and more significant scores than those living with a partner, in terms of anxiety, coinciding with the results of Newman and Anderson [71] and Bastida-González et al. [66].

As regards (h5), women are more resilient than men, it is not endorsed. Greater resilience has been known to help people overcome or face the negative effects of stress more successfully [72]. Previous studies have shown a higher prevalence of females showing higher resilience [73,74], which is counter to the data from this study, in which males showed higher levels of resilience than females. Other research has shown greater resilience in males [75], coinciding with the data obtained in this study. This fact comes to demonstrate the variability of results in the studies, the fruit of the multiple aspects that influence anxiety, not yet isolated in its totality. In addition, no significant differences in gender or resilience were found in the study of Cortés [76]. Other studies that have focused exclusively on resilience in adult females have established that the self-concept of resilience in females is clearly influenced by the nature of the situation currently causing the anxiety, as well as by the history of trauma [77]. It is evident in our study, in which females showed higher levels of anxiety in all dimensions and lower values of resilience.

Coinciding with the study of Cortés [76], previous studies have considered resilience as a moderating and protective variable against anxiety [77,78,79] or as a stress-compensating variable in risky environments, mitigating the symptoms of anxiety [80], a circumstance that is corroborated in this study.

Engagement has been studied in relation to sociodemographic variables, such as age and gender [81,82], with no relevant associations having been found. Although, higher levels of engagement were associated with males and lower levels were associated with females, as the present study showed, where men get higher engagement scores than women. In engagement studies, men and women have shown improved mental health [83], influencing their personal psychological and spiritual well-being [84], as a person who is perceived competent in practice could have a more positive attitude, thereby finding himself or herself more motivated.

In relation to (h6), optimism is positively related to resistance, it is confirmed. As for the non-existence of significant differences between males and females with respect to LOT-R, the finding was consistent with the past literature [85,86]. In addition, OAs who were physically active scored clearly and significantly higher on the optimism and LOT-R traits, the latter finding of which coincided with Garcia and Angulo [87].

Optimism has been found to become a highly useful protector against demoralization when the expected results are not adequate, in addition to strengthening and contributing to resilience by reinforcing it [88]. In the present study, higher scores in resilience were associated with higher values in LOT-R. In addition, optimists have been reported to have better physical and mental health as well as fewer episodes of anxiety and depression [89], both of which were supported in this study through the significant and negative association between LOT-R and anxiety.

Sex and age had no impact on optimism. The data corroborate previous studies, although it is true that the moderating effects of certain variables, such as PA and resilience, a multifactorial construct, should be analyzed in greater depth.

## 5. Conclusions

Some of the results of the study coincided with those of previous research. However, the study raised novel questions about gender and age differentiation in the OA group. Sports practice reduces anxiety, improves optimism, engagement and resilience. The results were relevant for proposing actions aimed at this target population, which has very marked characteristics, which are accentuated in most cases, for a large number of associated problems or situations.

In spite of the contributions, there were some limitations to the study. Firstly, the data consisted of solely self-reports, which are commonly used, but can include biases in the participants’ responses, exacerbate common variance and artificially increase correlations between variables [90]. In addition, the sample was comprised of only Spanish adults, which may have biased the results to the culture of the country. It would be important to replicate this type of study in other cultural contexts before implementing the results in the public policies of other countries.

It should be noted that the data showed that female OAs reached higher levels of A-T. The male participants in the sample under study were more optimistic and had higher levels of engagement and resilience. Age correlated positively with A-T, so the older participants were more likely to have higher A-T. The practice of PA, living with a partner and living indoors showed low values in A-T in the sample under study. Optimism was negatively associated with anxiety, such that higher values of optimism were associated with lower levels of anxiety and higher levels of resilience. Meanwhile, the opposite was true for resilience, with higher values of optimism being associated with higher values of resilience, a buffer element indicated positive psychology.

## Figures and Tables

**Table 1:** Means, standard deviations, reliability and correlations of the scales.

	Mean (*SD*)	Cronbach’s Alpha	1	2	3	4	5	6	7	8	9	10	11
TOTAL_Anxiety (A-T)	33.23 (9.63)	0.828	1										
Somatic_Anxiety (A-S)	9.59 (3.88)	0.802	0.863 **	1									
Concern_Anxiety (A-C)	13.33 (3.8)	0.831	0.736 **	0.455 **	1								
Deconcentration_Anxiety (A-D)	10.38 (4.17)	0.818	0.838 **	0.652 **	0.366 **	1							
LOT_TOTAL (LOT-T)	14.62 (3.65)	0.823	−0.317 **	−0.330 **	−0.113 *	−0.322 **	1						
LOT_Optimism (LOT-O)	8.64 (2.46)	0.808	−0.269 **	−0.257 **	−0.091	−0.309 **	0.780 **	1					
LOT_Pessimism (LOT-P)	6.17 (2.46)	0.830	0.272 **	0.291 **	0.125 *	0.246 **	−0.762 **	−0.170 **	1				
UWES_Total (UWES-T)	4.31 (1.08)	0.813	−0.260 **	−0.276 **	0.01	−0.347 **	0.378 **	0.436 **	−0.116 *	1			
UWES_Vigor (UWES-T)	4.28 (1.23)	0.806	−0.260 **	−0.298 **	0.019	−0.333 **	0.336 **	0.399 **	−0.109 *	0.925 **	1		
UWES_Dedication (UWES-D)	4.39 (1.16)	0.792	−0.218 **	−0.217 **	0.021	−0.317 **	0.376 **	0.443 **	−0.112 *	0.926 **	0.801 **	1	
UWES_Absorption (UWES-A)	4.25 (1.16)	0.800	−0.238 **	−0.242 **	−0.013	−0.305 **	0.329 **	0.359 **	−0.098	0.901 **	0.735 **	0.751 **	1
Resilience	27.28 (7.65)	0.813	−0.221 **	−0.223 **	−0.014	−0.287 **	0.458 **	0.551 **	−0.119 *	0.622 **	0.574 **	0.594 **	0.542 **

* *p* < 0.05; ** *p* < 0.01.

**Table 2:** Descriptive and comparative data for scales by sex.

	Sex		Average Difference	Student *t*		D
	Male (55.1%)	Female (44.9%)		*t* (379)	*p*-Value	
**Anxiety**						
A-T	31.91 (8.99)	34.30 (10.02)	−2.39	−2.422	0.016	−0.27
A-S	9.05 (3.84)	10.04 (3.85)	−0.99	−2.494	0.013	−0.28
A-C	13.04 (3.63)	13.57 (3.92)	−0.53	−1.344	0.18	−0.15
A-D	9.82 (3.92)	10.84 (4.32)	−1.01	−2.366	0.018	−0.27
**LOT**						
LOT-R	14.73 (3.58)	14.54 (3.71)	0.19	0.502	0.616	0.06
LOT-O	8.70 (2.32)	8.60 (2.57)	0.10	0.397	0.692	0.04
LOT-P	6.09 (2.39)	6.23 (2.52)	−0.15	−0.574	0.567	−0.06
**UWES**						
UWES-T	4.35 (1.02)	4.27 (1.14)	0.09	0.781	0.435	0.09
UWES-V	4.34 (1.20)	4.23 (1.26)	0.10	0.807	0.42	0.09
UWES-D	4.41 (1.06)	4.36 (1.23)	0.05	0.417	0.677	0.05
UWES-A	4.31 (1.09)	4.20 (1.22)	0.11	0.92	0.358	0.10
**Resilience**	27.74 (6.94)	26.90 (8.18)	0.84	1.064	0.288	0.12

A-T: Total Anxiety; A-S: Somatic Anxiety; A-C: Concern Anxiety; A-D: Deconcentration Anxiety; LOT-T: Lot Total; LOT-O: LOT Optimism; LOT-P: LOT Pessimism; UWES-T: UWES Total; UWES-D: UWES Dedication; UWES-A: UWES Absorption.

**Table 3:** Descriptive and comparative data for scales according to engagement in physical activity.

	Age	
	R	*p*-Value
Anxiety		
A-T	0.287	<0.001
A-S	0.216	0.001
A-C	0.059	0.252
A-D	0.341	<0.001
LOT		
LOT-R	−0.031	0.552
LOT-O	−0.042	0.408
LOT-P	0.007	0.894
UWES		
UWES-T	−0.082	0.11
UWES-V	−0.063	0.218
UWES-D	−0.055	0.285
UWES-A	−0.208	0.015
**Resilience**	0.001	0.991

A-T: Total Anxiety; A-S: Somatic Anxiety; A-C: Concern Anxiety; A-D: Deconcentration Anxiety; LOT-T: Lot Total; LOT-O: LOT Optimism; LOT-P: LOT Pessimism; UWES-T: UWES Total; UWES-D: UWES Dedication; UWES-A: UWES Absorption.

**Table 4:** Descriptive and comparative scale scores according to age.

	Age, Mean (*SD*)			ANOVA		*eta* ^2^
	<65	65–75	>75	F(2;377)	*p*-Valor	
Anxiety						
A-T	31.26 (8.51) a	33.51 (9.49) b	36.44 (11.00) c	7.948	< 0.001	0.04
A-S	9.13 (3.38) a	9.52 (4.07) ab	10.61 (4.23) b	3.89	0.021	0.02
A-C	13.13 (3.76)	13.48 (3.73)	13.43 (4.01)	0.342	0.711	0.002
A-D	9.05 (3.36) a	10.54 (4.10) b	12.56 (4.71) c	20.618	< 0.001	0.099
LOT						
LOT-R	14.53 (3.58)	14.74 (3.57)	14.59 (3.96)	0.127	0.881	0.001
LOT-O	8.66 (2.55)	8.67 (2.31)	8.55 (2.57)	0.069	0.933	0
LOT-P	6.21 (2.38)	6.09 (2.55)	6.24 (2.48)	0.134	0.875	0.001
UWES						
UWES-T	4.35 (1.03)	4.38 (1.04)	4.08 (1.24)	2.163	0.116	0.011
UWES-V	4.32 (1.19)	4.36 (1.20)	4.06 (1.33)	1.721	0.18	0.009
UWES-D	4.42 (1.11)	4.43 (1.12)	4.23 (1.31)	0.982	0.376	0.005
UWES-A	4.31 (1.10) a	4.34 (1.07) a	3.97 (1.38) b	3.069	0.048	0.016
Resilience	27.07 (7.54)	27.42 (7.17)	27.43 (8.73)	0.1	0.905	0.001

*SD*: standard deviation. *eta*^2^: effect size. a–c: two-to-two column comparisons. Between two different columns letters indicate statistically significant differences (Bonferroni correction). A-T: Total Anxiety; A-S: Somatic Anxiety; A-C: Concern Anxiety; A-D: Deconcentration Anxiety; LOT-T: Lot Total; LOT-O: LOT Optimism; LOT-P: LOT Pessimism; UWES-T: UWES Total; UWES-D: UWES Dedication; UWES-A: UWES Absorption.

**Table 5:** Effects of demographic variables and scales on LOT-R scores.

	*B* (ET)	Beta	T	*p*-Value
**Sex (Male, Female)**	0.24 (0.34)	0.033	0.708	0.479
Age	0.01 (0.02)	0.03	0.63	0.529
**Residence (Littoral, Inland)**	−0.51 (0.36)	−0.068	−1.406	0.161
**Centre type**				
None				
Private	−0.51 (0.63)	−0.04	−0.809	0.419
Public	−0.64 (0.38)	−0.081	−1.669	0.096
**Physical activity (Yes, No)**	0.31 (0.38)	0.039	0.801	0.424
**UWES**	0.36 (0.21)	0.107	1.686	0.093
**Anxiety**	−0.08 (0.02)	−0.206	−4.129	<0.001
**Resilience**	0.16 (0.03)	0.336	5.643	<0.001

*B*: non-standardized regression coefficient; *ET*: typical error; Beta: standardized regression coefficient.
